# Modulators of histone demethylase JMJD1C selectively target leukemic stem cells

**DOI:** 10.1002/2211-5463.13054

**Published:** 2020-12-16

**Authors:** Yong Yang, Xinjing Zhang, Xiaoyan Zhang, Yishu Wang, Xintong Wang, Linda Hu, Yao Zhao, Haihua Wang, Zhanju Wang, Haiying Wang, Lin Wang, Wilhelm G. Dirks, Hans G. Drexler, Xin Xu, Zhenbo Hu

**Affiliations:** ^1^ Laboratory for Stem Cell and Regenerative Medicine & Clinical Research Center The Affiliated Hospital of Weifang Medical University China; ^2^ Department of Anesthesiology Zibo Central Hospital China; ^3^ The Department of Obstetrics and Gynecology The Affiliated Hospital of Weifang Medical University China; ^4^ Beijing Beike Deyuan Bio‐Pharm Technology Co. Ltd China; ^5^ Upstate Medical University Syracuse NY USA; ^6^ The Department of Hematology the Affiliated Hospital of Weifang Medical University China; ^7^ The School of Physics and Optoelectronic Engineering Weifang University China; ^8^ Department of Human and Animal Cell Culture Leibniz‐Institute DSMZ‐German Collection of Microorganisms and Cell Cultures Braunschweig Germany; ^9^ School of Life Science and Technology Weifang Medical University China

**Keywords:** histone demethylases, JMJD1B, JMJD1C, leukemic stem cells, small molecular compounds

## Abstract

Leukemic stem cells (LSCs) comprise a very rare cell population that results in the development of acute myeloid leukemia. The selective targeting of drivers in LSCs with small molecule inhibitors holds promise for treatment of acute myeloid leukemia. Recently, we reported the identification of inhibitors of the histone lysine demethylase JMJD1C that preferentially kill *MLL* rearranged acute leukemia cells. Here, we report the identification of jumonji domain modulator #7 (JDM‐7). Surface plasmon resonance analysis showed that JDM‐7 binds to JMJD1C and its family homolog JMJD1B. JDM‐7 did not significantly suppress cell proliferation in liquid cell culture at higher doses, although it led to a significant decrease in semi‐solid colony formation experiments at lower concentrations. Moreover, low doses of JDM‐7 did not suppress the proliferation of erythroid progenitor cells. We identified that JDM‐7 downregulates the LSC self‐renewal gene *HOXA9* in leukemia cells. We further found that the structure of JDM‐7 is similar to that of tadalafil, a drug approved by the US Food and Drug Administration. Molecular docking and surface plasmon resonance analysis showed that tadalafil binds to JMJD1C. Moreover, similar to JDM‐7, tadalafil suppressed colony formation of leukemia cells in semi‐solid cell culture at a concentration that did not affect primary umbilical cord blood cells. In summary, we have identified JDM‐7 and tadalafil as potential JMJD1C modulators that selectively inhibit the growth of LSCs.

## Graphical abstract

JMJD1C is required for the maintenance of both leukemia cells and leukemic stem cells, serving as a potential therapeutic target of acute myeloid leukemia. JDM‐7 (jumonji domain modulator #7) and a structurally similar compound, tadalafil, a drug approved by the US Food and Drug Administration, are able to bind to JMJD1C, resulting in strong attenuation of leukemic stem cells and mild attenuation of leukemia cells, thereby repressing acute myeloid leukemia.

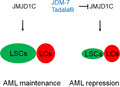

**Abbreviations**
AML: acute myeloid leukemia
CFU: colony‐forming units
FDA: Food and Drug Administration
IC_50_: half maximal inhibitory concentration
JDM‐7: jumonji domain modulator #7
LSC: leukemic stem cell
MNC: mononuclear cell
PDE5: phosphodiesterase type 5
SPR: surface plasmon resonance

Leukemic stem cells (LSCs) comprise a very rare cell population that exclusively reults in the development of acute myeloid leukemia (AML) [1, 2]. LSCs are characterized by a long resting phase, a tendency to chemotherapeutic resistances and the ability to mediate high recidivism rates. Recently, specific gene signatures of LSCs have been identified in which cell surface markers such as CD25, CD32, CD47, CD123, TIM‐3 and CXCR4 [2, 3, 4, 5, 6], as well as signaling pathways such as WNT/β‐catenin [7] or kinases such as HCK [2], are involved. Very important in this context was the finding that epigenetically modulating proteins are involved in the maintenance of LSCs and thus represent new and promising targets for the LSC‐specific therapy of AML. A selective eradication of LSCs would be of enormous therapeutic benefit for patients suffering from AML.

The first identified histone H3‐lysine‐4‐demethylase, LSD1, was found to be essential for maintaining the oncogenic potential and differentiation blockade of *MLL‐AF9* LSCs [8] as a result of the activity of its Jumonji domain as the catalytic center. The loss or repression of LSD1 by knockout experiments or using pharmaceutical inhibitors revealed a targeted killing effect on LSCs at the same time as protecting physiologically normal mononuclear cells (MNCs) isolated from umbilical cord blood, although there was a fatal effect on the development of erythroid progenitor cells [8]. The inhibition of the (H3K9)‐demethylase JMJD1C, on the other hand, causes only minor defects with respect to blood homeostasis and has a minor influence on the self‐renewal of the hematopoietic stem cells with a simultaneous reduction of LSC frequency in *MLL‐AF9*‐ and *HOXA9*‐driven leukemias [9]. Indeed, when the combined performance of the depletion of multiple chromatin‐associated genes on *MLL‐AF9* cells and normal c‐Kit^+^ bone marrow was considered, JMJD1C ranked first because the loss of JMJD1C led to the relatively strongest depletion of *MLL‐AF9* leukemia but the relatively lowest depletion of c‐Kit^+^ bone marrow [10].

Recently, we have reported the identification of JMJD1C inhibitors that preferentially kill *MLL* rearranged acute leukemia cells [11]. Here, we show that jumonji domain modulator #7 (JDM‐7) suppressed the colony‐forming units (CFU) of leukemia cells in semi‐solid methylcellulose culture, acting as a new potential JMJD1C modulator, whereas, at a similar concentration in suspension culture, JDM‐7 showed no significant inhibition of the growth of leukemia cells. Structurally related tadalafil also suppressed the CFU of leukemia cells, although both of the compounds do not inhibit MNCs obtained normal umbilical cord blood. In summary, we have identified new JMJD1C inhibitors that are able to target LSCs in AML.

## Results

### Identification of JDM‐7

**Fig. 1:**
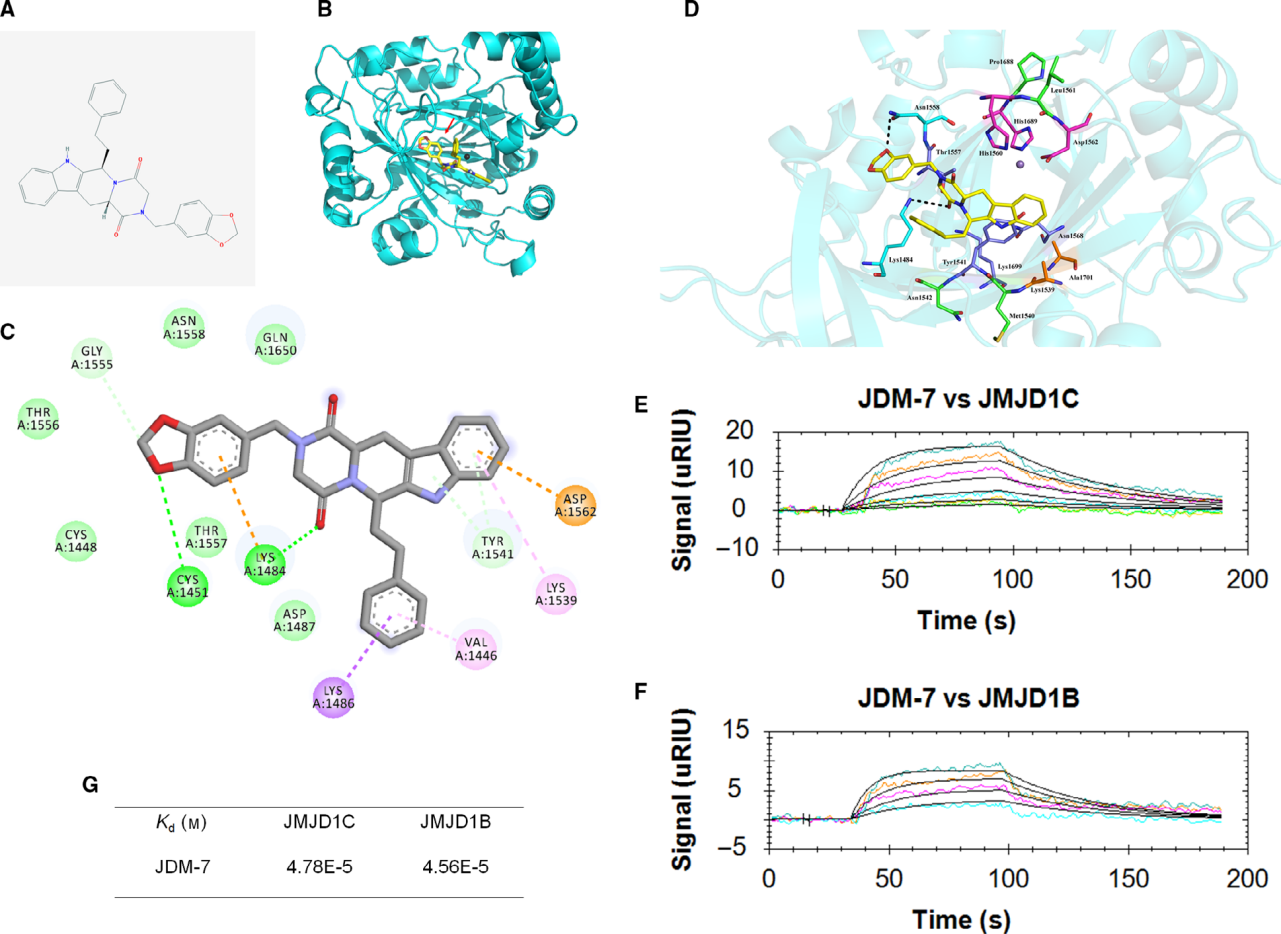
The identification of potential JMJD1C modulators JDM‐7. (A) The 2D molecular formula of JDM‐7 is shown. (B) Docking between JDM‐7 and the jumonji domain of JMJD1C (PDB ID 5FZO_A) is shown. The black ball represents Fe^2+^. The red arrow marks JDM‐7. (C, D) 2D and 3D binding modes were shown as indicated. For 3D binding modes, yellow ball‐and‐stick models represent compounds, purple particles represent Mn^2+^ and brown particles represent Fe^2+^; magenta ball‐and‐stick models represent residues bound by ions; navy blue ball‐and‐stick models represent residues bound by cofactors; pale brown ball‐and‐stick models represent peptide sites binding residues; green ball‐and‐stick models represent differential residues between JMJD1C and JMJD1B; sky blue ball‐and‐stick models represent non‐essential residues of JMJD1C and JMJD1B binding to compounds. Interaction was indicated with black lines. The docking modes were created using pymol (www.pymol.org). (E, F) SPR measurements of the binding between JDM‐7 and JMJD1C/JMJD1B. The sensorgrams of JDM‐7 binding to the chip‐immobilized partial JMJD1C and or JMJD1B proteins are expressed in RU (response unit) versus the time after subtracting the control signal. Recombinant partial JMJD1C and JMJD1B proteins are described in the Materials and methods. The JDM‐7 concentrations were 0.312, 0.625, 1.25, 2.5, 5.0 and 10 μm (from bottom to top). Colour lines: SPR data from different concentrations of the analytes; black lines: model fits. (G) The calculated dissociation constants (*K*_d_). All data shown are representative of at least two (*n* = 2) independent experiments.

We recently reported the identification of potential JMJD1C modulators [11] among which one compound (#7) with a β‐carbolin backbone attracted our attention (Fig. 1A‐D). In the first step to demonstrate specificity, we performed surface plasmon resonance (SPR) analysis to investigate the interaction between compound #7 and JMJD1C. As shown in Fig. 1E‐G and Video S1, compound #7 binds moderately to JMJD1C and JMJD1B at a concentration of 47.8 and 45.6 μm, respectively, such that we refer to compound #7 as JDM‐7.

### Selective repression of colony formation but not cell proliferation by JDM‐7

**Fig. 2:**
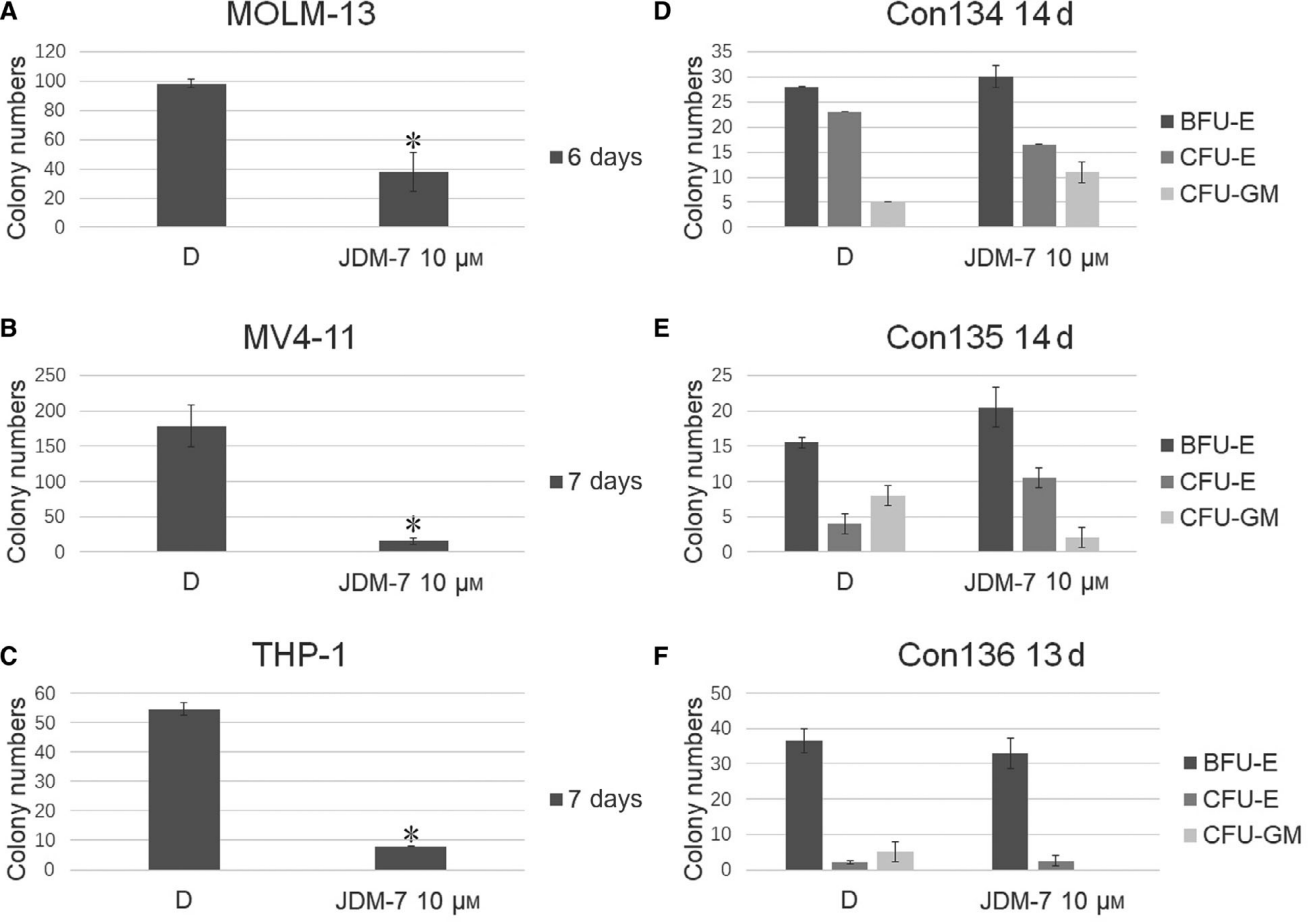
Identification of JDM‐7 (#7) as LSCs specific repressor. The effect of JDM‐7 on colony formation of *MLL*r AL cell lines MOLM‐13, MV4‐11, THP‐1 and cord blood cells. One thousand MOLM‐13, 2000 MV4‐11 cells and 2000 THP‐1 cells were cultured in methylcellulose medium (H4100; Stemcell Technologies) plus 10% fetal bovine serum with or without JDM‐7 for the days and compound concentrations indicated. Twenty‐five thousand cord blood cells were cultured in methylcellulose medium (H4434; Stemcell Technologies) with or without JDM‐7 for the days and compound concentrations indicated. Colonies were counted. Three (*n* = 3) replicated experiments were performed, a two‐sided Student’s *t*‐test was used for statistical analysis and *P* < 0.05 was considered statistically significant. Error bars indicate the SD. *P < 0.05. BFU, burst‐forming unit‐erythroid; CFU‐E, colony‐forming unit‐erythroid; CFU‐GM, colony‐forming unit‐granulocyte, macrophage.

The effect of JDM‐7 was first tested in model cultures of AML cell lines and no uniform effect was observed. As shown in Table 1, half maximal inhibitory concentration (IC_50_) values varied with respect to inhibiting the cell proliferation of multiple cell lines, although a concentration of at least 16 μm was required for proliferation inhibition. To demonstrate a specific effect, the impact of JDM‐7 was tested further in colony forming assays on AML cell lines and on primary MNCs isolated from umbilical cord blood. Therefore, we used the CFU read out of the semi‐solid methylcellulose culture system to determine the effects of JDM‐7. Unexpectedly, JDM‐7 can significantly suppress the CFU of most of the AML cell lines tested at 10 μm (Fig. 2A‐C and Fig. S1), whereas it had no suppressive effect on the CFU of MNCs isolated from umbilical cord blood, even in the treatments using JDM‐7 at a concentration of 25 μm (Figs 2D‐F and Fig. S1).

**Table 1:** The growth inhibitory IC_50_ of JDM‐7 and tadalafil on multiple cell lines. Leukemia cell lines listed were maintained as described in the Materials and methods. Cells were seeded at 30 000 mL^–1^ in 100 μL of medium in V‐bottom 96‐well plates and different concentrations of compounds (from 0.1 to 50 μm) were added 24 h after seeding. Four days later, cells were measured for cell proliferation using ATP detection kit as described in the Materials and methods. Three independent experiments were performed. IC_50_ was calculated using prism, version 5 (GraphPad Software Inc., San Diego, CA, USA).

Genetic alteration	Cell type	Cell line	JDM‐7 IC_50_ (4 days)	Tadalafil IC_50_ (4 days)
MLL‐AF4	AML	MV4‐11	18.43 ± 2.273	39.8 ± 3.721
MLL‐AF4	B‐ALL	SEM	> 50	> 100
MLL‐AF9	AML	MOLM‐13	19.54 ± 1.556	> 100
MLL‐AF9	AML	THP‐1	> 50	> 100
MYC AMP	AML	HL‐60	> 50	> 100
P53 mutation	T‐ALL	JURKAT	16.38 ± 1.287	73.4 ± 2.562
BCR‐ABL1	CML	K562	16.80 ± 2.107	> 100
NRAS mutation	AML	KG‐1	> 50	> 100
P53 mutation	AML	MUTZ‐8	41.02 ± 3.245	85.03 ± 5.082
PML‐RARA	AML	NB4	> 50	> 100

### JDM‐7 increases H3K9‐me1/2 demethylation and affects LSC signature genes

**Fig. 3:**
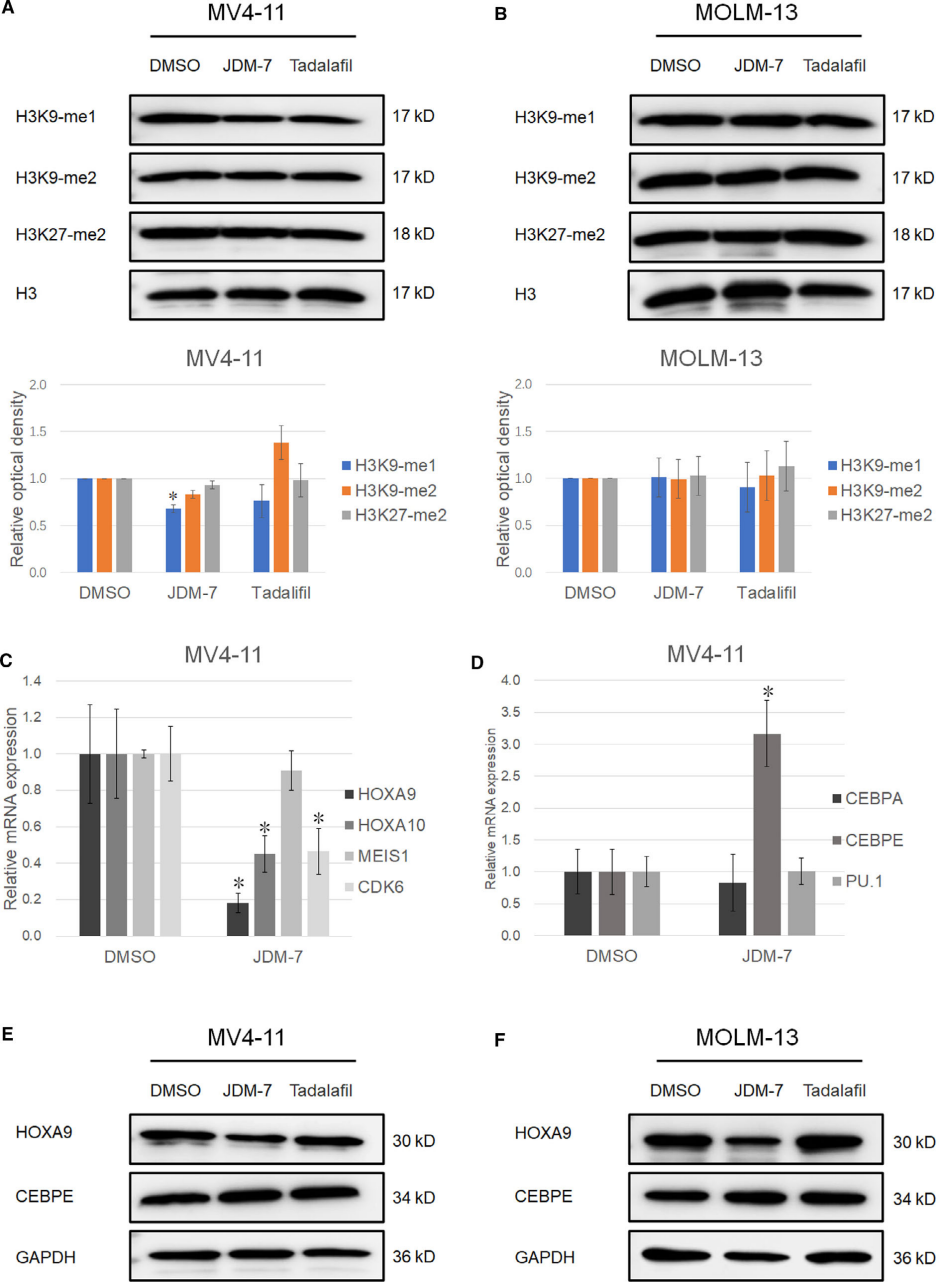
JDM‐7 and tadalafil increase H3K9‐me1/2 demethylation, downregulate HOXA9 and upregulate CEBPE in leukemia cells. (A, B) The effect of JDM‐7 and tadalafil on H3K9‐me1/2 in MV4‐11 (A) and MOLM‐13 (B) cells. 0.25 × 10^6^‧mL^–1^ cells were seeded and IC_50_ concentrations of compounds for cells (20 μm JDM‐7 and 40 μm tadalafil for MV4‐11; 20 μm JDM‐7 and 100 μm tadalafil for MOLM‐13) were added followed by incubation for 72 h. Cells were then collected for protein extraction and western blotting. A histogram showing the statistical results of three independent repeats is shown in (A) and (B). Three (*n* = 3) replicated experiments were performed, a two‐sided Student’s *t*‐test was used for statistical analysis and *P* < 0.05 was considered statistically significant. Error bars the SD. *P < 0.05. (C) The effect of JDM‐7 on the mRNA expression of HOXA9, HOXA10, MEIS1 and CDK6 in MV4‐11. 0.5 × 10^6^‧mL^–1^ MV4‐11 cells were seeded and, 24 h later, 10 μm JDM‐7 was added followed by incubation for 48 h. Cells were then collected for RNA extraction and quantitative PCR measurement of the expression of the indicated genes. (D) The effect of JDM‐7 on the mRNA expression of CEBPA, CEBPE and PU.1 in MV4‐11. 0.5 × 10^6^‧mL^–1^ MV4‐11 cells were seeded and, 24 h later, 10 μm JDM‐7 was added followed by incubation for 48 h. Cells were then collected for RNA extraction and quantitative PCR measurement of the expression of the indicated genes. For (C) and (D), three (*n* = 3) replicated experiments were performed, a two‐sided Student's *t*‐test was used for statistical analysis and *P* < 0.05 was considered statistically significant. Error bars indicate the SD. *P < 0.05. (E, F) The effect of JDM‐7 and tadalafil on HOXA9 and CEBPE in MV4‐11 (E) and MOLM‐13 (F) cells. 0.25 × 10^6^‧mL^–1^ cells were seeded and IC_50_ concentrations of compounds for cells (20 μm JDM‐7 and 40 μm tadalafil for MV4‐11; 20 μm JDM‐7 and 100 μm tadalafil for MOLM‐13) were added followed by incubation for 72 h. Cells were then collected for protein extraction and western blotting.

To uncover the mechanism of JDM‐7, *in vivo* H3K9 methylation levels were measured in AML cell lines treated with JDM‐7. As shown in Fig. 3A,B, JDM‐7 treatment decreases H3K9‐me1/2 levels at IC_50_ concentrations related to proliferation repression for MV4‐11 but not MOLM‐13. By contrast, H3K27‐me2 levels do not change with JDM‐7 treatment in MV4‐11.

To detect LSC signature genes such as *HOXA9*, *HOXA10*, *MEIS1* and *CDK6* [12], which are involved in the self‐renewal of LSCs, we next performed a quantitative PCR. As shown in Fig. 3C, JDM‐7 can repress the transcription of the LSC maintenance genes, except *MEIS1*, which showed a non‐significant reduction of less than 5% for specific transcripts. By contrast to the downregulation of *HOX* genes and *CDK6* gene expression, further RNA expression analysis showed a three‐fold upregulation of *CEBPE* at the mRNA level in the leukemia cell line MV4‐11, whereas the expression of additional myeloid differentiation marker *CEBPA* and *PU.1* was not affected by JDM‐7 (Fig. 3D). We further selected significantly downregulated *HOXA9* and upregulated *CEBPE* by JDM‐7 for western blotting. As shown in Fig. 3E,F, JDM‐7 decreases HOXA9 protein levels and increases CEBPE protein levels.

### The JDM‐7 analog tadalafil also represses LSCs

**Fig. 4:**
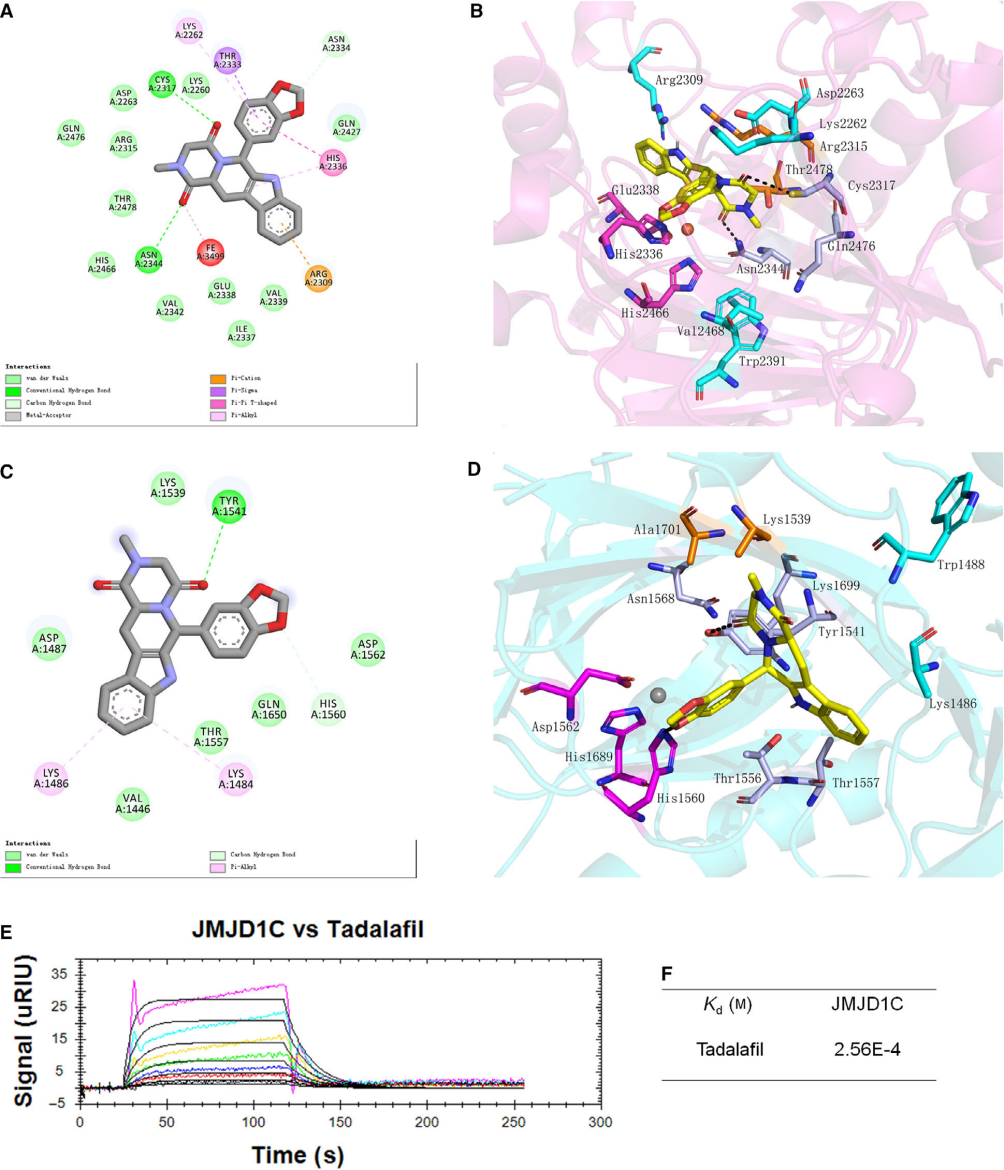
The identification of tadalafil as JDM‐7 analog. 2D and 3D binding modes of tadalafil and JMJD1C (A, B) or JMJD1B (C, D) are shown as indicated. For 3D binding modes, yellow ball‐and‐stick models represent compounds, purple particles represent Mn^2+^ and brown particles represent Fe^2+^; magenta ball‐and‐stick models represent residues bound by ions; navy blue ball‐and‐stick models represent residues bound by cofactors; pale brown ball‐and‐stick models represent peptide sites binding residues; green ball‐and‐stick models represent differential residues between JMJD1C and JMJD1B; sky blue ball‐and‐stick models represent non‐essential residues of JMJD1C and JMJD1B binding to compounds. An interaction was indicated with black lines. The docking modes were created using pymol (www.pymol.org). (E) SPR measurements of the binding between JMJD1C and tadalafil. The sensorgrams of tadalafil binding to the chip‐immobilized partial JMJD1C proteins are expressed in RU (response unit) versus time after subtracting the control signal. Recombinant partial JMJD1C proteins are described in Materials and methods. The tadalafil concentrations were from 7.86 × 10E with a two‐fold increase to 5.00 × 10E4 μm (from bottom to top). Colour lines, SPR data from different concentrations of the analytes; black lines, model fits. (F) The calculated dissociation constants (*K*_d_). All data shown are representative of at least two (*n* = 2) independent experiments.

**Fig. 5:**
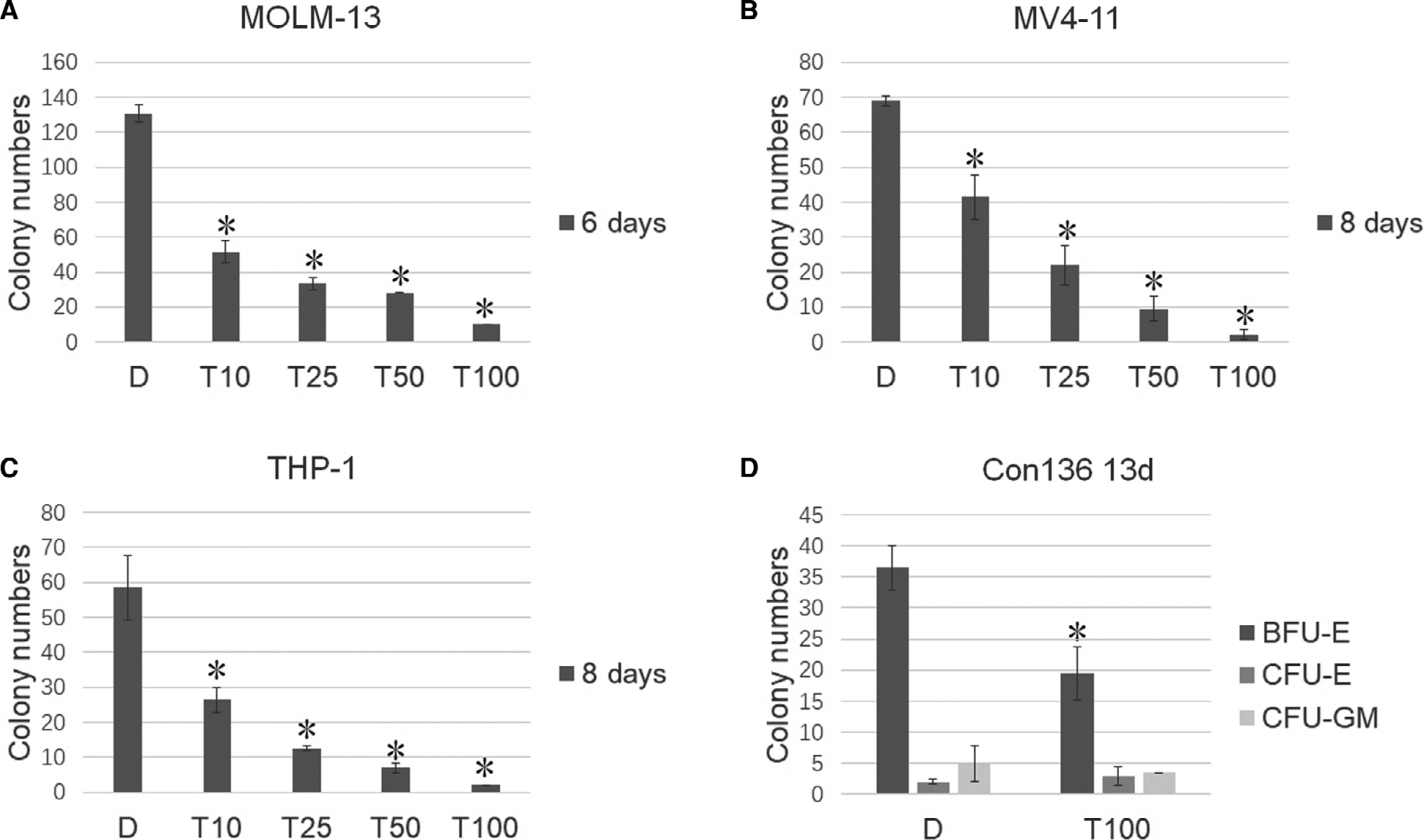
Tadalafil represses the CFU of leukemia cells. The effect of tadalafil on colony formation of *MLL*r AL cell lines MOLM‐13, MV4‐11, THP‐1 and cord blood cells. One thousand MOLM‐13, 2000 MV4‐11 cells and 2000 THP‐1 cells were cultured in methylcellulose medium (H4100; Stemcell Technologies) plus 10% fetal bovine serum with or without tadalafil for the days and compound concentrations indicated. Twenty‐five thousand cord blood cells were cultured in methylcellulose medium (H4434; Stemcell Technologies) with or without tadalafil for the days and compound concentrations indicated. Colonies were counted. Three (*n* = 3) replicated experiments were performed, a two‐sided Student's *t*‐test was used for statistical analysis and *P* < 0.05 was considered statistically significant. Error bars indicate the SD. *P < 0.05. T10, T25, T50 and T100 indicates tadalafil at 10, 25, 50 and 100 μm. BFU, burst‐forming unit‐erythroid; CFU‐E, colony‐forming unit‐erythroid; CFU‐GM, colony‐forming unit‐granulocyte, macrophage.

We searched for similar compounds to JDM‐7 and realized, unexpectedly, that tadalafil, a drug approved by the US Food and Drug Administration (FDA), showed strong structural similarity to JDM‐7. After virtual screenings and computational analysis using SPR, we found that tadalafil showed weaker binding of JMJD1C compared to JDM‐7 (Fig. 4 and Table 2). We next repeated cell proliferation and CFU assays to measure the impact of tadalafil on LSCs. As shown in Table 1, tadalafil was able to repress growth of AML leukemia cell lines at a very high concentration, although tadalafil also reduced the CFUs of leukemic cells by 90% at relatively lower concentrations (Fig. 5A‐C and S2). Most importantly, tadalafil did confirm specificity for LSCs because the repression of BFU‐E of primary MNCs from umbilical cord blood was reduced by only half (Figs 5D and S2). Similar to JDM‐7, tadalafil at IC_50_ concentrations of proliferation repression for the corresponding cell line also resulted in decreased H3K9‐me1/2 levels (Fig. 3A), downregulated *HOXA9* (Fig. 3E) and upregulated *CEBPE* (Fig. 3F).

**Table 2:** Docking between JDM‐7 and the jumonji domain of JMJD1C (PDB ID 5FZO _A) or JMJD1B (PDB ID 4C8D_A) is shown. The docking modes were created using pymol (www.pymol.org). The detailed information has been described previously [11].

Score (kcal·mol^−1^)	5FZO and JDM‐7	5FZO and tadalafil	4C8D and JDM‐7	4C8D and tadalafil
Pose 1	−7.8	−7.1	−6.7	−6.8
Pose 2	−7.4	−7.1	−6.3	−6.4
Pose 3	−7.4	−6.0	−6.3	−6.3
Pose 4	−5.8	−5.6	−6.2	−6.0
Pose 5	−5.3	−5.1	−6.2	−5.6
Pose 6	−5.0	−4.8	−6.2	−5.5
Pose 7		−4.1	−6.0	−5.4
Pose 8		–	−6.0	−5.3
Pose 9		–	−5.8	−5.2

## Discussion

LSCs are mostly resistant to conventional chemotherapy and their dormant state and location in a protective bone marrow niche is likely to be one of the critical components contributing to chemotherapy resistance. Many efforts have been made to find reagents specifically targeting LSCs. Antibodies targeting specific cell surface markers are an important method of eliminating LSCs. For example, monoclonal antibodies have been developed against CD44, CD123 and CD47, which showed good activity against AML LSCs in xenotransplantation models [13].

Small molecules offer more versatile possibilities for LSC‐specific targeting and can be tested using high‐throughput platforms [14]. A successful strategy using thioridazine could be identified that targets LSCs specifically as an antagonist of the dopamine receptor [15]. Silico‐screenings of publicly available gene expression databases identified further compounds that eradicate LSCs [16]. Moreover, the survival supporting proteins that have also been explored for the eradication of LSCs via BCL‐2 inhibition were found to target oxidative phosphorylation and selectively kill quiescent human LSCs [17].

In the present study, we report the identification of the JMJD1C modulator JDM‐7, which shows its effect on high cell densities not only at high concentrations, but also at low concentrations in colony‐forming tests. It is important to note that the inhibitory effect was only observed in LSCs, whereas primary MNCs from umbilical cord blood were only inhibited with much higher doses of JDM‐7. Interestingly, tadalafil, an erectile dysfunction drug approved by the FDA in 2003 and a benign prostatic hyperplasia drug approved by the FDA in 2011, is a structural analogue of JDM‐7. Corresponding experiments have confirmed that tadalafil has an apoptotic effect that is also specifically directed against LSCs.

Because current cytotoxic agents do not have a specific effect in the treatment of leukemias and do not spare physiologically normal erythropoiesis cells, new strategies are required that target the malignant stem cell population in a specific and preferred manner. JDM‐7 increases the histone demethylation levels *in vivo* and could further show its agonistic function in AML cell lines.

We have demonstrated that JDM‐7 increases the histone demethylation levels *in vivo*. JDM‐7 may be an agonist rather than an antagonist of JMJD1C/JMJD1B. For example, resveratrol, a polyphenol found in wines and considered to harbor major health benefits, was found to be an agonist of SIRT1 as a result of facilitating the binding between SIRT1 and its substrates [18].

JDM‐7, along with JDI‐4/12/16 identified by us [11], is designed based on the crystal structure of JMJD1C (5FZO), but not JMJD1B (4C8D) for which the enzymatic domain shows 57% similarity to JMJD1C. However, SPR analysis shows that JDM‐7 binds to JMJD1C and JMJD1B similarly, reflecting that conserved key residues in the jumonji domains of JMJD1C and JMJD1B may determine the affinities.

However, we did not consider JMJD1B as a mediator of the anti‐LSC effects of JDM‐7 because we previously showed that JMJD1B is a potential myeloid leukemia tumor suppressor [19]. Moreover, JMJD1C is expressed to a greater extent in *MLL*‐rearranged AML and is indispensable for the maintenance of *MLL*‐rearranged AML [10, 20], whereas JMJD1B is relatively lower in *MLL*‐rearranged AML [21]. Accordingly, JMJD1C is more likely mediator of JDM‐7.

Tadalafil, aside from its use in erectile dysfunction and benign prostatic hyperplasia, was shown to be able to repress malignant tumors in varying mechanisms. Tadalafil could directly inhibit colon and thyroid tumor cells [22, 23]. Tadalafil also synergizes with cyclooxygenase‐2 inhibitors to kill parental glioma and stem‐like glioma cells [24]. Moreover, tadalafil could also suppress head and neck squamous cell carcinomas and multiple myelomas by activating tumor immunity through repressing myeloid‐derived suppressor cells [25, 26, 27, 28, 29]. Although phosphodiesterase type 5 (PDE5) was reportedly responsible for the effect of tadalafil against tumors, tadalafil may also confer tumor repression by targeting JMJD1C/JMJD1B. On the other hand, JDM‐7 may also target PDE5 for LSC inhibition because JDM‐7 binds to PDE5 more strongly than tadalafil, as indicated by our molecular docking (Table S1).

## Conclusions

In summary, we have identified JDM‐7 and tadalafil that specifically kill LSCs by targeting JMJD1C. Both JDM‐7 and tadalafil could synergize with routine chemotherapies and targeted therapies to enable the better killling of AML cells. In addition, because tadalafil is an FDA‐approved drug and its safety has been broadly investigated, its clinical usage for AML along with other anti‐leukemia drugs merits further investigation.

## Materials and methods

### Reagents, human cell lines and primary samples

Compound JDM‐7 was purchased from Topscience (Shanghai, China). Tadalafil was purchased from Sigma (Shanghai, China).

The human cell lines and cord blood cells used in the present study have been described previously [11]. MV4‐11, SEM, MOLM‐13, THP‐1, HL‐60, JURKAT, K562, KG‐1, MUTZ‐8 and NB4 are maintained by the Leibniz‐Institute DSMZ‐German Collection of Microorganisms and Cell Cultures (Braunschweig, Germany). Cells were cultured using the recommended culture conditions (https://www.dsmz.de). All cells have been authenticated using the standard short tandem repeats genotyping method (ANSI/ATCC ASN‐0002‐2011) [30]. All experiments were performed with mycoplasma‐free cells.

Umbilical cord blood cells of healthy newborns (*n* = 3) collected in China were provided by the Affiliated Hospital of Weifang Medical University and Weifang People's Hospital. Informed written consent was obtained from parents of all newborns in accordance with the Declaration of Helsinki, and the study was approved by the Ethics committee of Weifang Medical University. Peripheral blood MNCs were isolated from healthy umbilical cord blood cells using a Ficoll method immediately after the samples were obtained. Freshly prepared cells were cultured in RPMI 1640 medium plus 10% fetal bovine serum (Life Technologies, Shanghai, China) and were viably frozen.

### SPR

The SPR analysis has been described previously [11]. Analyses of small molecular binding and binding kinetics were performed at 25 °C on a BIAcore S200 SPR instrument (GE Healthcare, Shanghai, China) and Reichert 4SPR instrument (Reichert Technologies, Depew, NY, USA). The 1.05 × PBS‐P (0.05% P20 in 1.05 × PBS) with 5% dimethylsulfoxide running buffer was prepared, filtered and degassed before use. Flow cells of CM7 sensor chip were activated for 7 min with a 1 : 1 mixture of 0.1 m *N*‐hydroxysuccinimide and 0.1 m *N*‐ethyl‐N9‐(3‐diethylaminopropyl)‐carbodiimide at a flow rate of 10 μL·min^−1^. Partial JMJD1B purified from *Escherichia coli* cells (Cusabio, Wuhan, China) and partial JMJD1C (2274–2498aa) purified from *E. coli* cells (Cusabio) were diluted in 10 mm sodium acetate (pH 4.0) to a concentration of 100 μg·mL^−1^ and immobilized on different flow cells to over 10 000 response units. The remaining binding sites on the chips were blocked by 1 m ethanolamine (pH 8.5) at a flow rate of 10 μL·min^−1^ for 7 min. The small molecules were injected at different indicated concentrations and passed over adjacent target and control flow cells at a flow rate of 30 μL·min^−1^ for 60 s. After 60 s of dissociation, the bound analytes were removed by a 20‐s wash with 50% dimethylsulfoxide in running buffer. The resulting data after subtracting the control values was analyzed by fitting to a 1 : 1 Langmuir binding model using the BIAcore S200 and Reichert 2SPR evaluation software. All data shown are representative of at least two independent experiments.

### Cell proliferation assay

The cell proliferation assay has been described previously [11]. The optimal cell seeding was determined empirically for all cell lines by examining the growth of a wide range of seeding densities in 96‐well V‐bottom plates aiming to identify the conditions that permitted exponential proliferation for 6 days. Cells were then plated at the optimal seeding density 24 h before treatment (in triplicate) with a multiple‐point two‐fold dilution series of compounds or 0.1% dimethylsulfoxide. Plates were incubated for 6 days at 37 °C in 5% CO_2_. Cells were then lysed with ViaLight Plus kit (Lonza, Cologne, Germany) in accordance with the manufacturer’s instructions and the chemiluminescence signal was detected with a Spark 10M microplate reader (Tecan, Crailsheim, Germany). For each cell line, the IC_50_ was determined from concentration‐dependence curves using spss, version 19 (IBM Corp., Armonk, NY, USA). The variance between the groups is statistically similar. All data shown are representative of three independent experiments.

### Colony formation assay

As reported previously [11], clonogenic potential was assessed through colony growth in the presence of dimethylsulfoxide (vehicle), JDM‐7 or tadalafil at concentrations as indicated. Human MOLM‐13, MV4‐11 and THP‐1 cells were plated in methylcellulose media (Methocult H4100; Stemcell Technologies, Cologne, Germany) supplemented with 10% fetal bovine serum in triplicates at a cell dose of 1000 per plate for MOLM‐13 and 2000 for MV4‐11 and THP‐1. Cord blood cells were plated in methylcellulose media (Methocult H4434; Stemcell Technologies) at a cell dose of 25 000 per plate. Cells were incubated at 37 °C in 5% CO_2_ for the indicated number of days, after which time colonies were counted. The variance between the groups is statistically similar. Three replicated experiments were performed. *P* < 0.05 was considered statistically significant.

### Western blotting

Cells were seeded at 0.25 million per mL and IC_50_ concentrations of compounds for cells (20 μm JDM‐7 and 40 μm tadalafil for MV4‐11; 20 μm JDM‐7 and 100 μm tadalafil for MOLM‐13) were added followed by incubation for 72 h. Cells were then collected for protein extraction and run on 10–15% SDS/PAGE gels for electrophoresis. Anti‐histone H3 (#06‐755; Millipore, Darmstadt, Germany), anti‐H3K9‐me1 (#07‐450; Millipore), anti‐H3K9‐me2 (ab1220; Abcam, Shanghai, China), anti‐H3K27‐me2 (ab24684; Abcam), anti‐HOXA9 (ab140631; Abcam) and anti‐CEBPE (ab172616; Abcam) antibodies were used for primary detection. As secondary antibodies, either anti‐rabbit or anti‐mouse IgG conjugated with horseradish peroxidase (GE Healthcare, Braunschweig, Germany) were used. Western Lightning Plus ECL (Perkin Elmer, Waltham, MA, USA) reagents were used for fluorescence production and Amersham Imager 600 (GE Healthcare, China) was used for fluorescence detection to visualize the proteins detected. The optical densities of the protein bands were analyzed using Amersham Imager 600 software. Three independent repeats were performed. Relative optical densities were calculated for the indicated bands by dividing the corresponding control bands. The control groups were set as 1 and treatment groups were calculated accordingly.

### Quantitative PCR

As described previously [11], cells were seeded at 0.5 million‧mL^–1^ and, 24 h later, were incubated with JDM‐7 for a further 48 h. Cells were then collected for RNA isolation. Two micrograms of total RNA from MV4‐11 were reverse transcribed into cDNA using Invitrogen Superscript II reverse transcriptase (Life Technologies) in accordance with the manufacturer's instructions. Random primers were used to obtain cDNA. Synthesized cDNA served as templates in 20‐µL quantitative PCR reactions. Quantitative PCR was performed using SYBR protocols (Takara, Dalian, China). The PCR was run in an ABI7500 fast real time PCR machine (Applied Biosystems, Foster City, CA, USA) with quantitative PCR cycling conditions of 95 °C for 30 s, followed by 40 cycles of 95 °C for 3 s and 60 °C for 30 s. Relative concentrations of each target template were calculated according to the comparative Ct method. Expression of target transcripts was standardized to glyceraldehyde‐3 phosphate dehydrogenase. Quantitative PCR analyses were performed in triplicate. The variance between the groups is statistically similar. Three independent experiments were performed and *P* < 0.05 was considered statistically significant. The primers used are available upon request.

### Statistical analysis

Quantitative results (cell proliferation assay, colony formation assay, quantitative PCR and western blotting) are reported as the mean ± SD. The statistical analysis was performed using a two‐sided Student's *t*‐test. *P* < 0.05 was considered statistically significant.

## Conflict of interest

The authors declare no conflict of interest.

## Author contributions

YY and ZXJ performed most of the experiments. ZXY took samples from healthy newborns and performed the cell proliferation assays. WYS performed the cell culture procedures. WXT performed the virtual screenings and molecular docking analyses. HL drafted the article. ZY and WHH conducted the quantitative PCR. WZJ and WHY took samples from AML patients and performed the cell proliferation assays. WL performed the statistical analysis. DGW and DGH genotyped cell lines. XX and HZB designed and supervised the project and finalized the paper.

## Supporting information


**Table S1.** Docking between PDE5 (3BJC) and JDM‐7/tadalafil. The docking modes were created using pymol (www.pymol.org). The detailed information has been described previously [1].
**Fig. S1.** The effect of JDM‐7 on colony formation of *MLL*r AL cell lines MOLM‐13, MV4‐11, THP‐1 and cord blood cells. One thousand MOLM‐13, 2000 MV4‐11 cells and 2000 THP‐1 cells were cultured in methylcellulose medium (H4100; Stemcell Technologies) plus 10% fetal bovine serum with or without JDM‐7 for the days and compound concentrations indicated. Twenty‐five thousand cord blood cells were cultured in methylcellulose medium (H4434; Stemcell Technologies) with or without JDM‐7 for the days and compound concentrations indicated. Colonies were counted. Three (*n* = 3) replicated experiments were performed and *P* < 0.05 was considered statistically significant. BFU, burst‐forming unit‐erythroid; CFU‐E, colony‐forming unit‐erythroid; CFU‐GM, colony‐forming unit‐granulocyte, macrophage. Scale bars = 100 μm.
**Fig. S2.** The effect of tadalafil on colony formation of *MLL*r AL cell lines MOLM‐13, MV4‐11, THP‐1 and cord blood cells. One thousand MOLM‐13, 2000 MV4‐11 cells and 2000 THP‐1 cells were cultured in methylcellulose medium (H4100; Stemcell Technologies) plus 10% fetal bovine serum with or without tadalafil for the days and compound concentrations indicated. Twenty‐five thousand cord blood cells were cultured in methylcellulose medium (H4434; Stemcell Technologies) with or without tadalafil for the days and compound concentrations indicated. Colonies were counted. Three (*n* = 3) replicated experiments were performed and *P* < 0.05 was considered statistically significant. BFU, burst‐forming unit‐erythroid; CFU‐E, colony‐forming unit‐erythroid; CFU‐GM, colony‐forming unit‐granulocyte, macrophage. Scale bars, 100 μm.
**Video S1.** Cartoons of docking between JDM‐7 and JMJD1C (5FZO)/JMJD1B (4C8D) are shown. Yellow ball‐and‐stick models represent compounds, purple particles represent Mn^2+^ and brown particles represent Fe^2+^; magenta ball‐and‐stick models represent residues bound by ions; navy blue ball‐and‐stick models represent residues bound by cofactors; pale brown ball‐and‐stick models represent peptide sites binding residues; green ball‐and‐stick models represent differential residues between JMJD1C and JMJD1B; sky blue ball‐and‐stick models represent non‐essential residues of JMJD1C and JMJD1B binding to compounds. An interaction is indicated with black lines. The docking modes were created using pymol (www.pymol.org).

## Data Availability

The data are available from the corresponding author upon appropriate request.
