# Supplementary material for: Modulators of histone demethylase JMJD1C selectively target leukemic stem cells
Source: FEBS Open Bio. 2020 Dec 16;11(1):265–77. doi: 10.1002/2211-5463.13054 (PMC7780120; doi:10.1002/2211-5463.13054)
Supplement: Supplementary file 1 — Table S1. Docking between PDE5 (3BJC) and JDM‐7/tadalafil. The docking modes were created using pymol (www.pymol.org). The detailed information has been described previously [1]. Fig. S1. The effect of JDM‐7 on colony formation of MLLr AL cell lines MOLM‐13, MV4‐11, THP‐1 and cord blood cells. One thousand MOLM‐13, 2000 MV4‐11 cells and 2000 THP‐1 cells were cultured in methylcellulose medium (H4100; Stemcell Technologies) plus 10% fetal bovine serum with or without JDM‐7 for the days and compound concentrations indicated. Twenty‐five thousand cord blood cells were cultured in methylcellulose medium (H4434; Stemcell Technologies) with or without JDM‐7 for the days and compound concentrations indicated. Colonies were counted. Three (n = 3) replicated experiments were performed and P < 0.05 was considered statistically significant. BFU, burst‐forming unit‐erythroid; CFU‐E, colony‐forming unit‐erythroid; CFU‐GM, colony‐forming unit‐granulocyte, macrophage. Scale bars = 100 μm. Fig. S2. The effect of tadalafil on colony formation of MLLr AL cell lines MOLM‐13, MV4‐11, THP‐1 and cord blood cells. One thousand MOLM‐13, 2000 MV4‐11 cells and 2000 THP‐1 cells were cultured in methylcellulose medium (H4100; Stemcell Technologies) plus 10% fetal bovine serum with or without tadalafil for the days and compound concentrations indicated. Twenty‐five thousand cord blood cells were cultured in methylcellulose medium (H4434; Stemcell Technologies) with or without tadalafil for the days and compound concentrations indicated. Colonies were counted. Three (n = 3) replicated experiments were performed and P < 0.05 was considered statistically significant. BFU, burst‐forming unit‐erythroid; CFU‐E, colony‐forming unit‐erythroid; CFU‐GM, colony‐forming unit‐granulocyte, macrophage. Scale bars, 100 μm. Video S1. Cartoons of docking between JDM‐7 and JMJD1C (5FZO)/JMJD1B (4C8D) are shown. Yellow ball‐and‐stick models represent compounds, purple particles represent Mn2+ and brown particles r [file FEB4-11-265-s001.zip › feb413054-sup-0003-Supinfo.doc]

**Supplementary Materials**

**Modulators of histone demethylase JMJD1C selectively target leukemia stem cells**

**Running title**: JMJD1C modulators target leukemia stem cells

^*^Yong Yang^1^, ^*^Xinjing Zhang^2^, Xiaoyan Zhang^3^, Yishu wang^1^, Xintong Wang^4^, Linda Hu^5^, Yao Zhao^1^, Haihua Wang^1^, Zhanju Wang^6^, Haiying Wang^6^, Lin Wang^7^, Wilhelm G. Dirks^8^, Hans G. Drexler^8^, Xin Xu^1,9^, and Zhenbo Hu^1^

^1^Laboratory for Stem Cell and Regenerative Medicine, The Affiliated Hospital of Weifang Medical University, ^2^Department of Anesthesiology, Zibo Central Hospital, Zibo, Shandong, China 255020, ^3^The Department of Obstetrics and Gynecology, Affiliated Hospital of Weifang Medical University, ^4^Beijing Beike Deyuan Bio-Pharm Technology Co.Ltd, Beijing 100094, China, ^5^Upstate Medical University, Syracuse, NY, USA 13210, ^6^ The Department of Hematology, the Affiliated Hospital of Weifang Medical University, ^7^The School of Physics and Optoelectronic Engineering, Weifang University, Weifang, Shandong, China 261061, ^8^Department of Human and Animal Cell Culture, Leibniz-Institute DSMZ-German Collection of Microorganisms and Cell Cultures, D-38124 Braunschweig, Germany, ^9^College of Bioscience and Technology, Weifang Medical University, Weifang, Shandong, China 261031

^*^These authors contributed equally to this work

**Corresponding authors**:

**Zhenbo Hu**, Ph.D. & MD, Email: huzhenbo@wfmc.edu.cn. Telephone: +86 536 2602261, Fax: +86 536 8650309.

**Xin Xu**, Ph.D., Email: xinxu@wfmc.edu.cn. Telephone: +86 536 2602261, Fax: +86 536 8650309.

**Contents:**

Supplemental Table 1

Supplemental Figure 1

Supplemental Figure 2

Supplementary Cartoon (.mpg) Legends

**Supplemental Table 1. The docking between PDE5 (3BJC) and JDM-7/Tadalafil**.

| **Score (Kcal/mol)** | **3BJC&Tadalafil** | **3BJC&JDM-7** |
| --- | --- | --- |
| **Pose 1** | -8.6 | -10.3 |
| **Pose 2** | -8.1 | -10.0 |
| **Pose 3** | -7.7 | -10.0 |
| **Pose 4** | -7.7 | -9.8 |
| **Pose 5** | -7.7 | -9.6 |
| **Pose 6** | -7.7 | -9.3 |
| **Pose 7** | -7.7 | -9.3 |
| **Pose 8** | -7.3 | -9.0 |
| **Pose 9** | -7.2 | -8.9 |

The docking between PDE5 (PDB ID 3BJC) and JDM-7/Tadalafil is shown. The docking modes were created using Pymol ([www.pymol.org/](http://www.pymol.org/)). The detailed information has been described previously [1].

1.	Xu X, Wang L, Hu L, Dirks WG, Zhao Y, Wei Z, et al. Small molecular modulators of JMJD1C preferentially inhibit growth of leukemia cells. Int J Cancer. 2020 Jan 15;146(2):400-412.

**Supplemental Figure 1. The effect of JDM-7 on colony formation of *MLL*r AL cell lines MOLM-13, MV4-11, THP-1, and cord blood cells**. 1000 MOLM-13, 2000 MV4-11 cells, and 2000 THP-1 cells were cultured in methylcellulose medium (H4100, Stemcell) plus 10% FBS with or without JDM-7 with days and compound concentrations as indicated. 25,000 cord blood cells were cultured in methylcellulose medium (H4434, Stemcell) with or without JDM-7 with days and compound concentrations as indicated. Colonies were counted. Three (n=3) replicated experiments were performed and P value smaller than 0.05 was regarded statistically significant. BFU, burst-forming unit-erythroid; CFU-E, colony-forming unit-erythroid; CFU-GM, colony-forming unit-granulocyte, macrophage. Scale bars, 100μm.

**Supplemental Figure 2. The effect of Tadalafil on colony formation of *MLL*r AL cell lines MOLM-13, MV4-11, THP-1, and cord blood cells**. 1000 MOLM-13, 2000 MV4-11 cells, and 2000 THP-1 cells were cultured in methylcellulose medium (H4100, Stemcell) plus 10% FBS with or without Tadalafil with days and compound concentrations as indicated. 25,000 cord blood cells were cultured in methylcellulose medium (H4434, Stemcell) with or without Tadalafil with days and compound concentrations as indicated. Colonies were counted. Three (n=3) replicated experiments were performed and P value smaller than 0.05 was regarded statistically significant. BFU, burst-forming unit-erythroid; CFU-E, colony-forming unit-erythroid; CFU-GM, colony-forming unit-granulocyte, macrophage. Scale bars, 100μm.

**Supplemental Cartoons.** **The cartoons of docking between JDM-7 and JMJD1C (5FZO)/JMJD1B (4C8D) is shown.** Yellow ball-and-stick models represent compounds; purple particles represent Mn^2+^ and brown particles represent Fe^2+^; magenta ball-and-stick models represent residues bound by ions; navy blue ball-and-stick models represent residues bound by cofactors; pale brown ball-and-stick models represent peptide sites binding residues; green ball-and-stick models represent differential residues between JMJD1C and JMJD1B; sky blue ball-and-stick models represent non-essential residues of JMJD1C and JMJD1B binding to compounds. Interaction was indicated with black lines. The docking modes were created using Pymol ([www.pymol.org/](http://www.pymol.org/)).
